# Bromido{*N*-methyl-*N*′-[1-(2-pyrid­yl)ethyl­idene]ethane-1,2-diamine-κ^3^ *N*,*N*′,*N*′′}­(thio­cyanato-κ*N*)­copper(II)

**DOI:** 10.1107/S1600536810027534

**Published:** 2010-07-17

**Authors:** Li-Jun Liu

**Affiliations:** a Experimental Center, Linyi Normal University, Linyi Shandong 276005, People’s Republic of China

## Abstract

In the title mononuclear copper(II) compound, [CuBr(NCS)(C_10_H_15_N_3_)], the Cu^II^ atom is five-coordinated by three N atoms of the Schiff base ligand, the N atom of a thio­cyanate ligand and by one bromide ion forming a distorted square-pyramidal geometry. In the crystal structure, mol­ecules are linked through inter­molecular N—H⋯Br hydrogen bonds into chains propagating along [101].

## Related literature

For general background to Schiff base–copper(II) complexes, see: Adhikary *et al.* (2009 ▶); Al-Karawi (2009 ▶); Xiao & Zhang (2009 ▶); Rajasekar *et al.* (2010 ▶); Sang & Lin (2010 ▶); Qin *et al.* (2010 ▶). For related copper complexes with square-pyramidal coordination, see: Wang *et al.* (2010 ▶); Zhang *et al.* (2009 ▶); Wei *et al.* (2007 ▶).
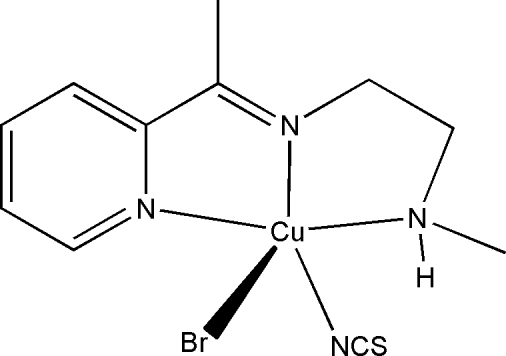


## Experimental

### 

#### Crystal data

[CuBr(NCS)(C_10_H_15_N_3_)]
*M* *_r_* = 378.78
Monoclinic, 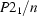

*a* = 10.979 (2) Å
*b* = 11.407 (2) Å
*c* = 12.001 (3) Å
β = 109.033 (2)°
*V* = 1420.8 (5) Å^3^
*Z* = 4
Mo *K*α radiation
μ = 4.48 mm^−1^
*T* = 298 K
0.30 × 0.27 × 0.27 mm

#### Data collection

Bruker APEXII CCD area-detector diffractometer
Absorption correction: multi-scan (*SADABS*; Sheldrick, 2004 ▶) *T*_min_ = 0.347, *T*_max_ = 0.377
8078 measured reflections
3022 independent reflections
1892 reflections with *I* > 2σ(*I*)
*R*_int_ = 0.070

#### Refinement

*R*[*F* ^2^ > 2σ(*F* ^2^)] = 0.053
*wR*(*F* ^2^) = 0.137
*S* = 0.97
3022 reflections
168 parameters
1 restraint
H atoms treated by a mixture of independent and constrained refinement
Δρ_max_ = 0.86 e Å^−3^
Δρ_min_ = −1.01 e Å^−3^

### 

Data collection: *APEX2* (Bruker, 2004 ▶); cell refinement: *SAINT* (Bruker, 2004 ▶); data reduction: *SAINT*; program(s) used to solve structure: *SHELXS97* (Sheldrick, 2008 ▶); program(s) used to refine structure: *SHELXL97* (Sheldrick, 2008 ▶); molecular graphics: *SHELXTL* (Sheldrick, 2008 ▶); software used to prepare material for publication: *SHELXTL*.

## Supplementary Material

Crystal structure: contains datablocks global, I. DOI: 10.1107/S1600536810027534/ci5132sup1.cif

Structure factors: contains datablocks I. DOI: 10.1107/S1600536810027534/ci5132Isup2.hkl

Additional supplementary materials:  crystallographic information; 3D view; checkCIF report

## Figures and Tables

**Table 1:** Selected bond lengths (Å)

Cu1—N4	1.949 (5)
Cu1—N2	1.965 (5)
Cu1—N1	2.019 (5)
Cu1—N3	2.044 (5)
Cu1—Br1	2.7228 (10)

**Table 2:** Hydrogen-bond geometry (Å, °)

*D*—H⋯*A*	*D*—H	H⋯*A*	*D*⋯*A*	*D*—H⋯*A*
N3—H3*A*⋯Br1^i^	0.90 (1)	2.69 (4)	3.494 (5)	150 (6)

Symmetry code: (i) 
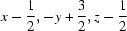
.

**Fig. 1.**
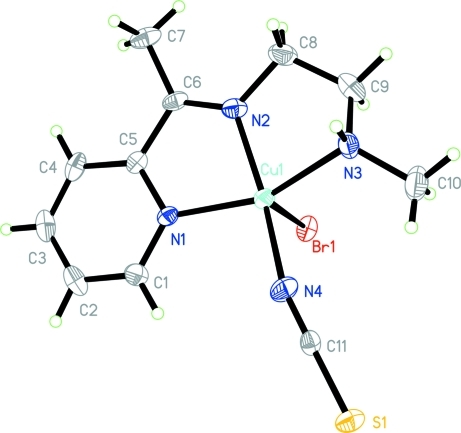
The molecular structure of the title complex with 30% probability displacement ellipsoids.

**Fig. 2.**
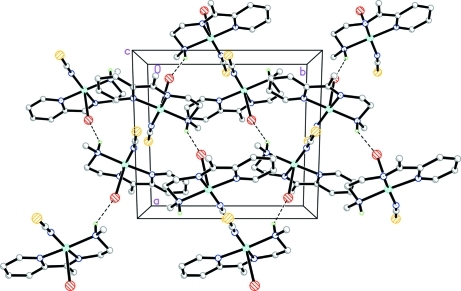
The crystal packing of the title complex, viewed along the c axis. Hydrogen bonds are shown as dashed lines.

## supplementary crystallographic information

### Comment

Copper(II) complexes with Schiff bases have been widely investigated in coordination chemistry and biological chemistry (Adhikary *et al.*, 2009; Al-Karawi, 2009; Xiao & Zhang, 2009; Rajasekar *et al.*, 2010; Sang & Lin, 2010; Qin *et al.*, 2010). In the present paper, the title new copper complex with the Schiff base ligand *N*methyl-*N'*-(1-pyridin-2-ylethylidene)ethane-1,2-diamine, is reported.

The Cu^II^ atom in the title complex (Fig. 1) is five-coordinated by one pyridine N, one imine N, and one amine N atoms of a Schiff base ligand, by one bromide atom, and by one N atom of a thiocyanate ligand, forming a square-pyramidal geometry. The bond lengths (Table 1) related to the Cu atom are comparable with those observed in similar copper complexes with square-pyramidal geometry (Wang *et al.*, 2010; Zhang *et al.*, 2009; Wei *et al.*, 2007).

In the crystal structure, molecules are linked through intermolecular N—H···Br hydrogen bonds (Table 2) to form chains running along the *a* axis (Fig. 2).

### Experimental

2-Acetylpyridine (0.1 mmol, 12.1 mg), ammonium thiocyanate (0.1 mmol, 7.6 mg), and copper bromide (0.1 mmol, 22.3 mg) were mixed and stirred in methanol (20 ml) at reflux for 2 h, to give a blue solution. The solution was cooled to room temperature, and blue block-shaped single crystals were formed by slow evaporation of the solution in air.

### Refinement

Atom H3A attached to N3 was located in a difference Fourier map and refined isotropically, with the N–H distance restrained to 0.90 (1) Å. The remaining H atoms were positioned geometrically (C–H = 0.93-0.97 Å) and refined using a riding model, with *U*_iso_(H) = 1.2*U*_eq_(C) and 1.5*U*_eq_(C_methyl_).

### Crystal data

**Table:**

[CuBr(NCS)(C_10_H_15_N_3_)]	*F*(000) = 756
*M**_r_* = 378.78	*D*_x_ = 1.771 Mg m^−^^3^
Monoclinic, *P*2_1_/*n*	Mo *K*α radiation, λ = 0.71073 Å
Hall symbol: -P 2yn	Cell parameters from 2570 reflections
*a* = 10.979 (2) Å	θ = 2.5–26.5°
*b* = 11.407 (2) Å	µ = 4.48 mm^−^^1^
*c* = 12.001 (3) Å	*T* = 298 K
β = 109.033 (2)°	Block, blue
*V* = 1420.8 (5) Å^3^	0.30 × 0.27 × 0.27 mm
*Z* = 4	

### Data collection

**Table:**

Bruker APEXII CCD area-detector diffractometer	3022 independent reflections
Radiation source: fine-focus sealed tube	1892 reflections with *I* > 2σ(*I*)
graphite	*R*_int_ = 0.070
ω scans	θ_max_ = 27.0°, θ_min_ = 2.2°
Absorption correction: multi-scan (*SADABS*; Sheldrick, 2004)	*h* = −13→14
*T*_min_ = 0.347, *T*_max_ = 0.377	*k* = −14→12
8078 measured reflections	*l* = −15→8

### Refinement

**Table:**

Refinement on *F*^2^	Primary atom site location: structure-invariant direct methods
Least-squares matrix: full	Secondary atom site location: difference Fourier map
*R*[*F*^2^ > 2σ(*F*^2^)] = 0.053	Hydrogen site location: inferred from neighbouring sites
*wR*(*F*^2^) = 0.137	H atoms treated by a mixture of independent and constrained refinement
*S* = 0.97	*w* = 1/[σ^2^(*F*_o_^2^) + (0.068*P*)^2^] where *P* = (*F*_o_^2^ + 2*F*_c_^2^)/3
3022 reflections	(Δ/σ)_max_ = 0.001
168 parameters	Δρ_max_ = 0.86 e Å^−^^3^
1 restraint	Δρ_min_ = −1.01 e Å^−^^3^

### Special details

**Table:**

Geometry. All esds (except the esd in the dihedral angle between two l.s. planes) are estimated using the full covariance matrix. The cell esds are taken into account individually in the estimation of esds in distances, angles and torsion angles; correlations between esds in cell parameters are only used when they are defined by crystal symmetry. An approximate (isotropic) treatment of cell esds is used for estimating esds involving l.s. planes.
Refinement. Refinement of F^2^ against ALL reflections. The weighted R-factor wR and goodness of fit S are based on F^2^, conventional R-factors R are based on F, with F set to zero for negative F^2^. The threshold expression of F^2^ > 2sigma(F^2^) is used only for calculating R-factors(gt) etc. and is not relevant to the choice of reflections for refinement. R-factors based on F^2^ are statistically about twice as large as those based on F, and R- factors based on ALL data will be even larger.

### Fractional atomic coordinates and isotropic or equivalent isotropic displacement parameters (Å^2^)

**Table:**

	*x*	*y*	*z*	*U*_iso_*/*U*_eq_	
Cu1	0.18062 (6)	0.60367 (6)	0.05737 (6)	0.0319 (2)	
Br1	0.38736 (6)	0.64845 (6)	0.25208 (5)	0.0412 (2)	
N1	0.2487 (4)	0.4486 (4)	0.0218 (4)	0.0292 (11)	
N2	0.2631 (5)	0.6533 (5)	−0.0577 (4)	0.0356 (12)	
N3	0.1033 (5)	0.7682 (4)	0.0253 (4)	0.0405 (13)	
N4	0.0715 (5)	0.5412 (5)	0.1429 (5)	0.0451 (14)	
S1	−0.03147 (18)	0.50606 (17)	0.32203 (16)	0.0522 (5)	
C1	0.2396 (6)	0.3440 (5)	0.0690 (5)	0.0390 (15)	
H1	0.1917	0.3382	0.1200	0.047*	
C2	0.2974 (7)	0.2460 (6)	0.0456 (7)	0.0527 (19)	
H2	0.2893	0.1748	0.0803	0.063*	
C3	0.3683 (7)	0.2534 (6)	−0.0307 (6)	0.0528 (19)	
H3	0.4090	0.1874	−0.0474	0.063*	
C4	0.3778 (6)	0.3601 (6)	−0.0815 (6)	0.0454 (17)	
H4	0.4249	0.3672	−0.1330	0.054*	
C5	0.3152 (5)	0.4572 (5)	−0.0541 (5)	0.0314 (13)	
C6	0.3174 (5)	0.5760 (5)	−0.1021 (5)	0.0331 (14)	
C7	0.3743 (7)	0.5979 (6)	−0.1975 (6)	0.0519 (18)	
H7A	0.3179	0.5672	−0.2706	0.078*	
H7B	0.4566	0.5597	−0.1780	0.078*	
H7C	0.3851	0.6806	−0.2052	0.078*	
C8	0.2442 (7)	0.7750 (6)	−0.0943 (6)	0.0518 (18)	
H8A	0.1787	0.7814	−0.1712	0.062*	
H8B	0.3238	0.8082	−0.0987	0.062*	
C9	0.2019 (7)	0.8386 (6)	−0.0024 (6)	0.0535 (19)	
H9A	0.2752	0.8504	0.0683	0.064*	
H9B	0.1669	0.9148	−0.0322	0.064*	
C10	0.0578 (8)	0.8231 (6)	0.1167 (6)	0.064 (2)	
H10A	0.1306	0.8425	0.1842	0.096*	
H10B	0.0030	0.7692	0.1395	0.096*	
H10C	0.0104	0.8931	0.0855	0.096*	
C11	0.0286 (5)	0.5262 (5)	0.2159 (5)	0.0312 (14)	
H3A	0.038 (5)	0.760 (6)	−0.043 (3)	0.080*	

### Atomic displacement parameters (Å^2^)

**Table:**

	*U*^11^	*U*^22^	*U*^33^	*U*^12^	*U*^13^	*U*^23^
Cu1	0.0334 (4)	0.0328 (4)	0.0350 (4)	0.0035 (3)	0.0184 (3)	0.0024 (3)
Br1	0.0369 (4)	0.0467 (4)	0.0389 (4)	0.0031 (3)	0.0108 (3)	−0.0090 (3)
N1	0.028 (3)	0.034 (3)	0.029 (3)	−0.001 (2)	0.014 (2)	0.000 (2)
N2	0.030 (3)	0.042 (3)	0.036 (3)	0.004 (2)	0.013 (2)	0.011 (2)
N3	0.049 (3)	0.030 (3)	0.043 (3)	0.007 (2)	0.014 (3)	−0.003 (2)
N4	0.039 (3)	0.056 (4)	0.050 (3)	0.000 (3)	0.028 (3)	0.000 (3)
S1	0.0599 (12)	0.0548 (11)	0.0570 (11)	−0.0083 (9)	0.0397 (10)	−0.0003 (9)
C1	0.041 (4)	0.036 (4)	0.040 (4)	−0.001 (3)	0.015 (3)	0.003 (3)
C2	0.052 (5)	0.029 (4)	0.071 (5)	0.001 (3)	0.012 (4)	−0.003 (3)
C3	0.055 (5)	0.040 (5)	0.057 (5)	0.006 (3)	0.009 (4)	−0.016 (4)
C4	0.039 (4)	0.058 (5)	0.042 (4)	0.006 (3)	0.017 (3)	−0.016 (3)
C5	0.026 (3)	0.039 (4)	0.028 (3)	0.001 (3)	0.008 (3)	−0.002 (3)
C6	0.023 (3)	0.050 (4)	0.029 (3)	0.002 (3)	0.013 (2)	0.007 (3)
C7	0.046 (4)	0.077 (5)	0.041 (4)	0.002 (4)	0.026 (3)	0.013 (4)
C8	0.056 (5)	0.046 (5)	0.066 (5)	0.006 (3)	0.036 (4)	0.020 (4)
C9	0.055 (5)	0.033 (4)	0.065 (5)	0.002 (3)	0.008 (4)	0.008 (3)
C10	0.079 (6)	0.060 (5)	0.054 (5)	0.026 (4)	0.022 (4)	−0.008 (4)
C11	0.022 (3)	0.029 (3)	0.044 (4)	0.000 (2)	0.012 (3)	−0.006 (3)

### Geometric parameters (Å, °)

**Table:**

Cu1—N4	1.949 (5)	C3—C4	1.380 (9)
Cu1—N2	1.965 (5)	C3—H3	0.93
Cu1—N1	2.019 (5)	C4—C5	1.398 (8)
Cu1—N3	2.044 (5)	C4—H4	0.93
Cu1—Br1	2.7228 (10)	C5—C6	1.476 (8)
N1—C1	1.338 (7)	C6—C7	1.494 (8)
N1—C5	1.343 (7)	C7—H7A	0.96
N2—C6	1.274 (7)	C7—H7B	0.96
N2—C8	1.451 (8)	C7—H7C	0.96
N3—C9	1.470 (9)	C8—C9	1.514 (9)
N3—C10	1.484 (8)	C8—H8A	0.97
N3—H3A	0.899 (10)	C8—H8B	0.97
N4—C11	1.135 (7)	C9—H9A	0.97
S1—C11	1.630 (7)	C9—H9B	0.97
C1—C2	1.359 (9)	C10—H10A	0.96
C1—H1	0.93	C10—H10B	0.96
C2—C3	1.384 (10)	C10—H10C	0.96
C2—H2	0.93		
			
N4—Cu1—N2	168.1 (2)	C3—C4—H4	120.6
N4—Cu1—N1	97.2 (2)	C5—C4—H4	120.6
N2—Cu1—N1	79.4 (2)	N1—C5—C4	121.3 (6)
N4—Cu1—N3	98.4 (2)	N1—C5—C6	114.4 (5)
N2—Cu1—N3	82.0 (2)	C4—C5—C6	124.3 (6)
N1—Cu1—N3	157.5 (2)	N2—C6—C5	113.7 (5)
N4—Cu1—Br1	95.75 (16)	N2—C6—C7	125.1 (6)
N2—Cu1—Br1	95.90 (14)	C5—C6—C7	121.1 (6)
N1—Cu1—Br1	94.79 (12)	C6—C7—H7A	109.5
N3—Cu1—Br1	99.67 (15)	C6—C7—H7B	109.5
C1—N1—C5	118.9 (5)	H7A—C7—H7B	109.5
C1—N1—Cu1	127.4 (4)	C6—C7—H7C	109.5
C5—N1—Cu1	113.6 (4)	H7A—C7—H7C	109.5
C6—N2—C8	125.1 (5)	H7B—C7—H7C	109.5
C6—N2—Cu1	118.7 (4)	N2—C8—C9	106.6 (5)
C8—N2—Cu1	115.8 (4)	N2—C8—H8A	110.4
C9—N3—C10	112.8 (5)	C9—C8—H8A	110.4
C9—N3—Cu1	104.6 (4)	N2—C8—H8B	110.4
C10—N3—Cu1	117.9 (4)	C9—C8—H8B	110.4
C9—N3—H3A	106 (5)	H8A—C8—H8B	108.6
C10—N3—H3A	111 (5)	N3—C9—C8	109.1 (5)
Cu1—N3—H3A	104 (5)	N3—C9—H9A	109.9
C11—N4—Cu1	160.6 (5)	C8—C9—H9A	109.9
N1—C1—C2	122.8 (6)	N3—C9—H9B	109.9
N1—C1—H1	118.6	C8—C9—H9B	109.9
C2—C1—H1	118.6	H9A—C9—H9B	108.3
C1—C2—C3	119.2 (7)	N3—C10—H10A	109.5
C1—C2—H2	120.4	N3—C10—H10B	109.5
C3—C2—H2	120.4	H10A—C10—H10B	109.5
C4—C3—C2	119.1 (6)	N3—C10—H10C	109.5
C4—C3—H3	120.5	H10A—C10—H10C	109.5
C2—C3—H3	120.5	H10B—C10—H10C	109.5
C3—C4—C5	118.7 (6)	N4—C11—S1	179.1 (6)

### Hydrogen-bond geometry (Å, °)

**Table:**

*D*—H···*A*	*D*—H	H···*A*	*D*···*A*	*D*—H···*A*
N3—H3A···Br1^i^	0.90 (1)	2.69 (4)	3.494 (5)	150 (6)

Symmetry codes: (i) *x*−1/2, −*y*+3/2, *z*−1/2.
